# Comparative Quantitative Analysis of Porcine Optic Nerve Head and Retina Subproteomes

**DOI:** 10.3390/ijms20174229

**Published:** 2019-08-29

**Authors:** Sebastian Funke, Carsten Schmelter, Sascha D. Markowitsch, Natarajan Perumal, Janis C. Heyne, Katharina Bell, Norbert Pfeiffer, Franz H. Grus

**Affiliations:** 1 Experimental and Translational Ophthalmology, University Medical Center, Johannes Gutenberg University Mainz, 55131 Mainz, Germany; 2 Department of Urology and Pediatric Urology, University Medical Center, 55131 Mainz, Germany

**Keywords:** optic nerve head, retina, glaucoma, *Sus scrofa domestica*, LC-MS, MALDI-TOF MS

## Abstract

Optic nerve head (ONH) and retina (RET) are the main sites of damage in neurodegenerative optic neuropathies including glaucoma. Up to date, little is known about the molecular interplay between these two adjoining ocular components in terms of proteomics. To close this gap, we investigated ONH and RET protein extracts derived from porcine eyes (*n* = 12) (*Sus scrofa domestica* Linnaeus 1758) using semi-quantitative mass spectrometry (MS)-based proteomics comprising bottom-up LC–ESI MS/MS and targeted SPE-MALDI-TOF MS analysis. In summary, more than 1600 proteins could be identified from the ONH/RET tissue complex. Moreover, ONH and RET displayed tissue-specific characteristics regarding their qualitative and semi-quantitative protein compositions. Gene ontology (GO)-based functional and protein–protein interaction analyses supported a close functional connection between the metabolic-related RET and the structural-associated ONH subproteomes, which could be affected under disease conditions. Inferred from the MS findings, stress-associated proteins including clusterin, ceruloplasmin, and endoplasmin can be proposed as extracellular mediators of the ONH/ RET proteome interface. In conclusion, ONH and RET show obvious proteomic differences reflecting characteristic functional features which have to be considered for future protein biomarker profiling studies.

## 1. Introduction

The optic nerve head (ONH) and the retina (RET) display distinct proteomic alterations associated with neurodegenerative diseases such as glaucoma. These alterations were found trough the analysis of original human material as well as ocular samples from animal models [1]. Especially, natural and induced disease models of the house swine (*Sus scrofa domestica* Linnaeus 1758) [2], including genetically modified animals [3], are promising systems for proteomic studies of neurodegenerative diseases [2,4,5,6], also considering ocular neuropathies such as glaucoma [7]. Humans and pigs share a high degree of similarity regarding neuronal anatomy and cognition [8]. This implies an important potential for porcine models in terms of research on human neurodegenerative diseases. The comparability of the porcine and human eye with respect to size, architecture, genomic and proteomic characteristics, as well as the appropriate material access, makes the pig attractive for investigations of ocular neuropathies via mass spectrometry (MS)-based proteomic platforms [9,10,11,12]. Despite the importance of porcine ocular models for studying neurodegenerative processes, so far, only few proteomic studies of the ocular material have been realized. The studies have so far focused on whole retinal tissues [13,14], specific isolated retinal layers [15], or particular cell types [16]. Although important for understanding molecular disease processes in the posterior eye region, including optic nerve diseases, proteomic investigations of the porcine ONH are still elusive. Up to date, and to the best of our knowledge, the comparison and characterization of ONH and RET subproteomes has not been realized. However, we believe that the proteomic analysis of the ONH/RET complex is essential to understand the crosstalk between these two specific regions of the eye, as both are highly vulnerable to neurodegenerative processes. We therefore performed a comparative proteomic study of porcine ONH and RET tissue samples using a state-of-the-art MS-based proteomics platform encompassing “bottom-up” high-performance liquid chromatography—electrospray ionization mass spectrometry (BU-LC–ESI MS/MS; termed shortly BULCMS) and targeted solid-phase extraction matrix-assisted laser desorption time-of-flight mass spectrometry (SPE-MALDI-TOF MS; termed shortly MB) analysis. Moreover, we used a targeted MS screening technology for the detection of the marker protein methyl-CpG-binding protein 2 (Gene name: *MECP2*) in RET and ONH tissue samples. Not only does MECP2 have an important function as transcriptional regulator and is able to interact with methylated DNA [17], but also it was found to be of lower abundance in human retinal tissues of glaucomatous donor eyes in comparison to non-glaucoma tissues [13]. Furthermore, the proper protein activity of MECP2 is essential for the function and viability of neuronal cells [18]. To understand the impact of MECP2 in the context of glaucoma or other neurodegenerative eye diseases, the investigation of the tissue-specific distribution of MECP2 in the posterior eye region is of high importance and will provide essential information about the molecular function as well as regulation of MECP2 in the porcine RET/ONH complex.

In addition, as there is still limited knowledge of the pig proteome despite the growing demand for proteomic data regarding this species for animal and biomedical research [19], the porcine ONH/RET protein catalogue represents an important contribution to the characterization of the “pig proteome” [4]. In particular, we believe that the resulting ONH/RET protein catalogue will provide a reference for proteome researchers focusing on porcine models of ocular neuropathies, e.g., glaucoma, taking tissue-specific protein distribution into consideration.

## 2. Results

**Figure 1:**
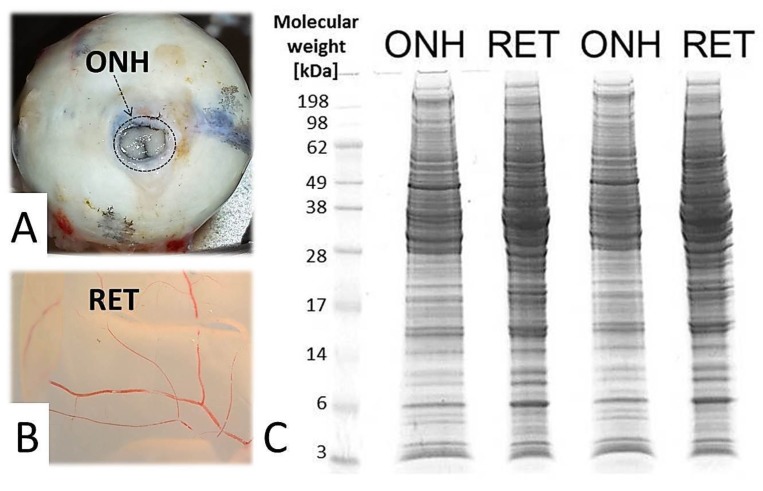
Sample preparation for comparative optic nerve head (ONH)/retina (RET) “bottom-up” high-performance liquid chromatography–electrospray ionization mass spectrometry BULCMS analysis. (**A**) Sample processing area (indicated by arrow and circle) of a porcine eye bulb corresponding to ONH tissue. (**B**) RET tissue isolated from the porcine eye bulb. (**C**) 1D SDS PAGE displaying ONH- and RET-specific migration pattern corresponding to the soluble tissue fraction. Gel lanes were used for tryptic in-gel digestion according to BULCMS analysis.

**Figure 2:**
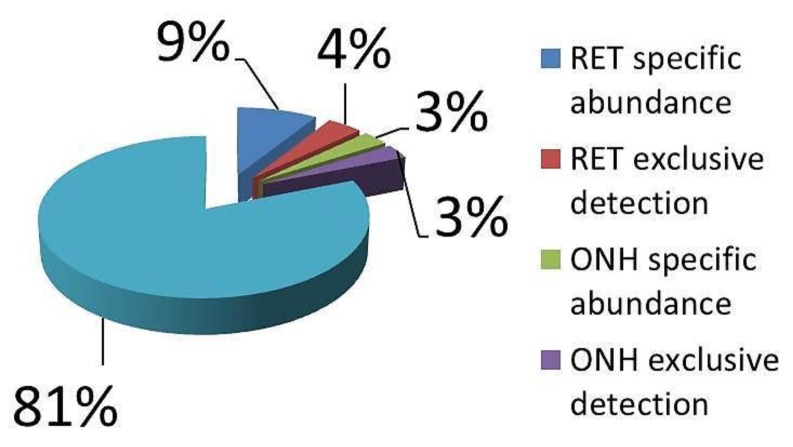
ONH/RET proteomic tissue distribution inferred from BULCMS analysis. Approximately 19% of recovered proteins display characteristic tissue abundances encompassing tissue-exclusive or abundance-related recovery.

**Figure 3:**
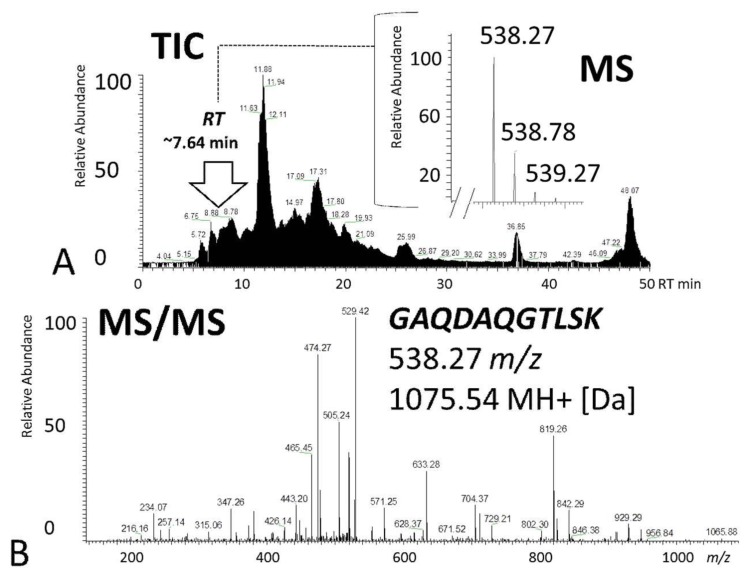
Exemplary identification of ONH-exclusive myelin basic protein (Gene name: *MBP*) corresponding to BULCMS analysis. (**A**) Total ion current (TIC) chromatogram including the monoisotopic peptide pattern of MBP-specific unique peptide at m/z 538.27 [1075.54 MH+ (Da), charge +2]. (**B**) MS/MS fragmentation spectrum of the MBP-specific peak at *m/z* 538.27 with the sequence *GAQDAQGTLSK*.

**Figure 4:**
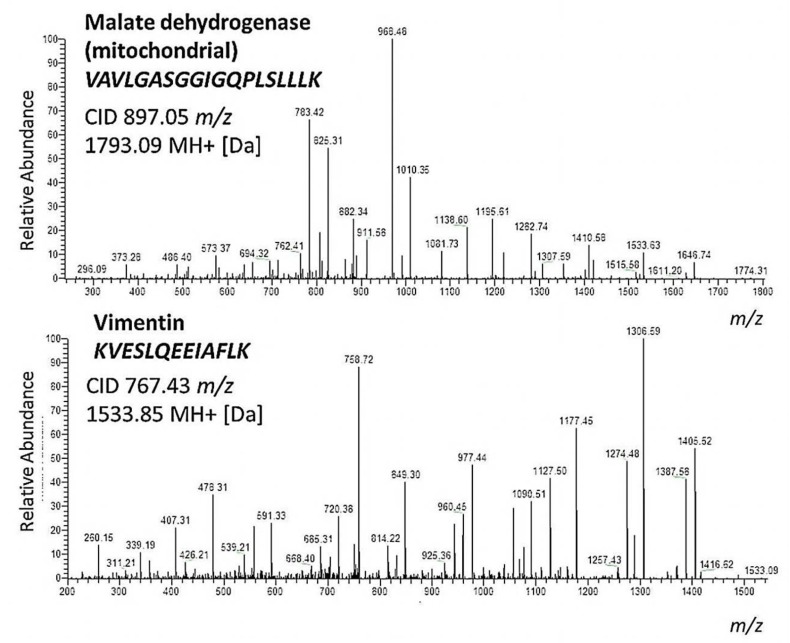
Exemplary BULCMS corresponding MS/MS fragmentation spectra leading to the identification of mitochondrial malate dehydrogenase (Gene name: *MDH2*), displaying RET-specific abundance, and vimentin (Gene name: *VIM*), showing ONH-specific abundance. The identification was based on MS/MS-spectra of the MDH2-specific peak at *m/z* 897.05 with the sequence *VAVLGASGGIGQPLSLLLK* and on tandem MS spectra of the VIM-specific peak at *m/z* 767.43 with the sequence *KVESLQEEIAFLK*.

**Figure 5:**
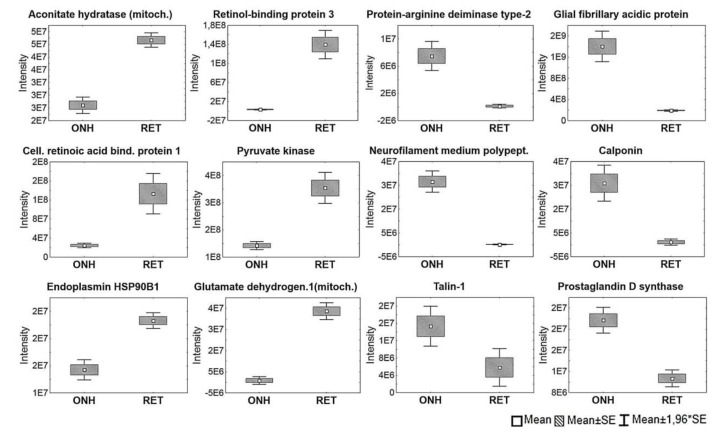
Exemplary candidate proteins characteristic for ONH or RET tissue. The illustrated label-free quantification (LFQ) protein abundance differences (*p* < 0.05) were based on BULCMS analysis followed by MaxQuant LFQ determination.

**Figure 6:**
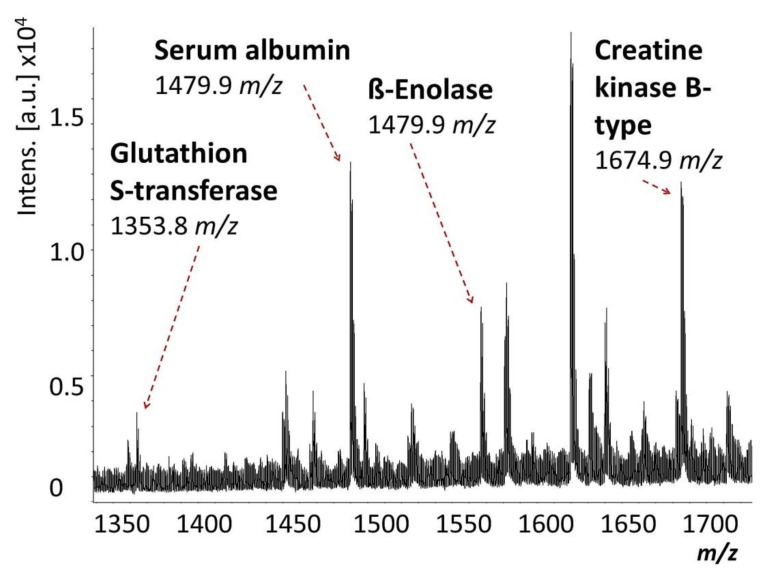
Exemplary solid-phase extraction matrix-assisted laser desorption time-of-flight (MB) MS spectrum displaying selected tissue candidate reporter peptide peaks.

**Figure 7:**
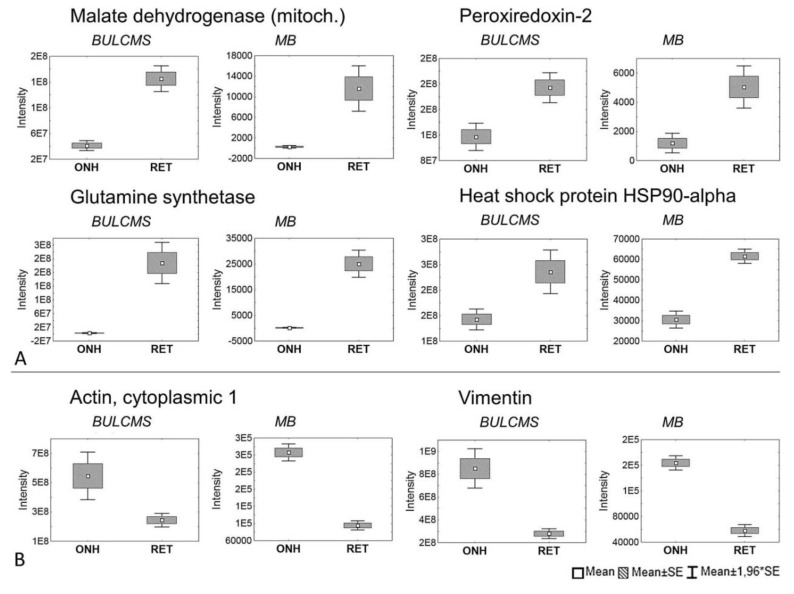
ONH/RET tissue-specific proteins determined by BULCMS and MB. (**A**) Exemplary metabolic enzymes and stress-related proteins show higher levels in RET than in ONH tissue (*p* < 0.05). (**B**) Exemplary structural proteins display ONH related elevated level abundances (*p* < 0.05).

**Figure 8:**
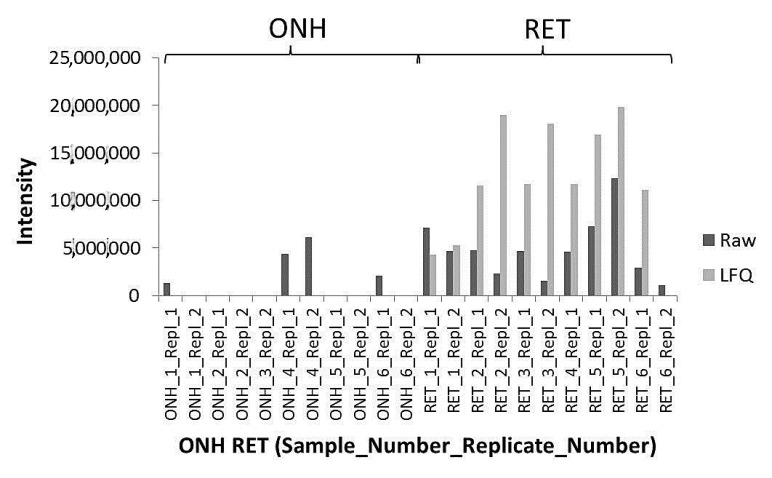
Targeted accurate inclusion mass screening (AIMS) LC–ESI-MS/MS profiling of MECP2 in the remaining ONH and RET tissue pellet samples demonstrating significantly higher MECP2 recovery rates from RET tissue compared to ONH tissue (*p* = 0.005898 [Raw], *p* = 0.000007 [LFQ]; Student’s *t*-test).

**Figure 9:**
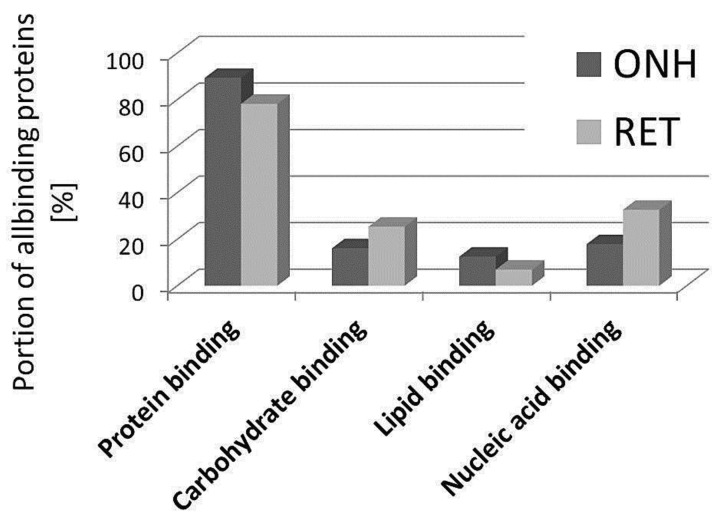
Molecular function determination of ONH/RET-specific proteins by gene ontology (GO) analysis. Most tissue-specific proteins display protein-binding features.

**Figure 10:**
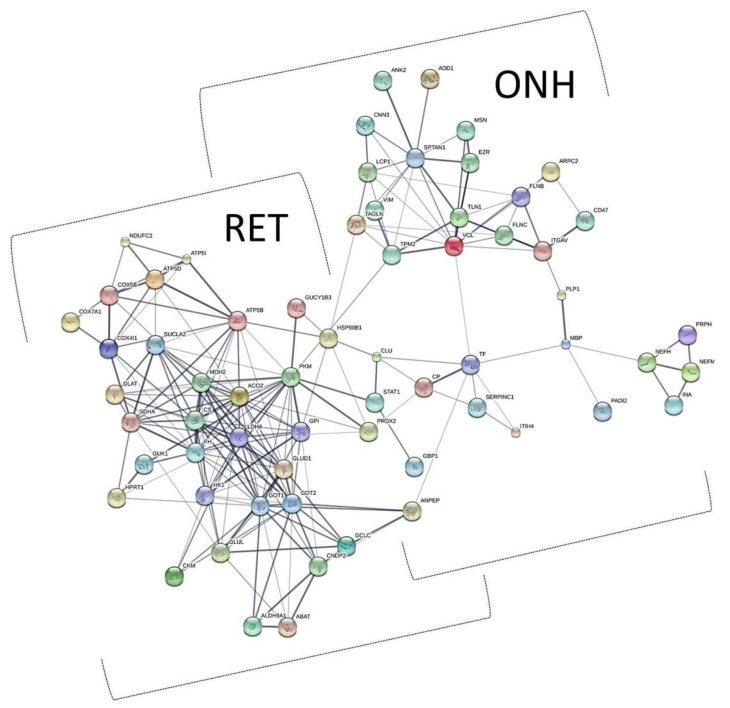
Protein-protein interaction (PPI) network analysis using STRING software. Tissue-specific candidates separate in two distinct interaction clusters, proposing a subproteomic interplay between RET metabolism and ONH structure. At key nodes between the two clusters, extracellular stress‑related proteins [e.g., endoplasmin (Gene name: *HSP90B1*), clusterin (Gene name: *CLU*), and serotransferrin (Gene name: *TF*)] can be found, suggestive of their mediating role between ONH structure and RET metabolism.

The 1D-SDS PAGE demonstrated characteristic protein patterns of ONH and RET samples (Figure 1). The protein pattern could be documented with high reproducibility in all four technical replicate gels, as determined by visual inspection. The BULCMS proteomic analysis of the RET/ONH complex led to the identification of 1659 proteins (FDR < 1%; see File S1). Regarding the ONH/RET protein catalogue, 60% of protein hits corresponded to uncharacterized protein species. Moreover, 81% of all identified proteins did not show significant quantitative differences between the two tissue types (*p* > 0.05), whereas approximately 19% of the remaining proteins displayed a tissue-specific distribution (*p* < 0.05) when comparing RET and ONH protein intensities (Figure 2). Thereby, approximately 72% of these tissue-specific proteins (19% of all identified proteins) were categorized as “RET-specific”, and 28% as “ONH-specific” (including proteins that showed a RAW and label-free quantification (LFQ) congruent intensity pattern, meaning identical “regulation” (*p* < 0.05), or exclusive protein detection indicated by zero RAW intensity values in RET or ONH). The biological significance of the determined proteomic tissue heterogeneity could be supported by the exclusive detection of axonal myelin sheet-related proteins in ONH samples, including myelin basic protein (Gene name: *MBP*), myelin proteolipid protein (Gene name: *PLP1*), and myelin-oligodendrocyte glycoprotein (Gene name: *MOG*) (ONH-exclusive MBP recovery is illustrated exemplarily in Figure 3). Top RET-specific detected proteins included the intracellular metabolic enzymes glutamate dehydrogenase 1 (Gene name: *GLUD1*), 6-phosphofructokinase (Gene name: *PFKL*), fumarate hydratase (Gene name: *FH*), fructose-bisphosphate aldolase (Gene name: *ALDOC*), guanylate kinase (Gene name: *GUK1*), aspartate aminotransferase (Gene name: *GOT1*), pyruvate kinase (Gene name: *PKM*), and aconitate hydratase (Gene name: *ACO2*). Moreover, retinol-binding protein 3 (Gene name: *RBP3*), cellular retinoic acid binding protein 1 (Gene name: *CRABP1*), medium-wave sensitive opsin (Gene name: *N/A*, “not annotated”), phosducin (Gene name: *PDC*) were significantly more abundant in RET samples, while interphotoreceptor retinoid binding protein (Gene name: *N/A*) as well as rhodopsin (Gene name: *RHO*) were exclusively detected in RET samples. The RET sample specific recovery of these characteristic retinal proteins supports the distinct proteomic tissue portraitures of the ONH and RET. Also, stress-related proteins, e.g., endoplasmin (Gene name: *HSP90B1*), heat shock 70 kDa protein 6 (Gene name*: HSPA6*), peroxiredoxin-2 (Gene name: *PRDX2*), and hypoxia up-regulated protein 1 (Gene name: *HYOU1*), were predominantly found in RET tissue. Synaptosomal-associated protein (Gene name: *SNAP25*) could exclusively be recovered from RET samples, in agreement with its presence in retinal photoreceptor and bipolar cell synapses [20]. ONH-specific proteins included talin-1 (Gene name: *TLN1*), protein arginine deiminase (type 2) (Gene name: *PADI2*), neurofilament medium polypeptide (Gene name: *NEFM*), neurofilament heavy polypeptide (Gene name: *NEFH*), calponin (Gene name: *CNN3*), transgelin (Gene name: *TAGLN*), vimentin (Gene name: *VIM*), as well as glial fibrillary acidic protein (F1RR02_PIG, Gene name: *GFAP*). Nerve tissue proteins prostaglandin D synthase (Gene name: *PGDS*), γ-synuclein (Gene name: *SNCG*), and ß-crystallin B1 (Gene name: *CRYBB1*) could be detected in both tissues, but with higher abundance in ONH. N-Myc downstream regulated gene 2 protein (Gene name: *NDRG2*), aquaporin 4 (Gene name: *AQP4*), and α-2-HS-glycoprotein (Gene name: *AHSG*) could be exclusively recovered from ONH samples. (Exemplary BULCMS spectra are illustrated in Figure 4; examples of RET/ONH protein distribution are given in Figure 5). The tissue-specific abundance could be validated by MB analysis (Figure 6; Figure 7; see File S1) for prominent RET-specific proteins (supplementary data) including malate dehydrogenase (Gene name: *MDH2*), HSP 90-α (Gene name: *HSP90A*), creatine kinase B-type (Gene name*: CKB*) and U-type (Gene name: *CKMT1*), glutamine synthetase (Gene name: *GLUL*), peroxiredoxin-2 (Gene name: *PRDX2*), S-arrestin (Gene name: *SAG*), clathrin (heavy chain 1) (Gene name: *CLTC*), glutathione S-transferase P (Gene name: *GSTP1*), as well as for the ONH-specific proteins vimentin (Gene name: *VIM*) and galectin-1 (Gene name: *LGALS1*). Regarding BULCMS analysis, especially glutamine synthetase (RET/ONH_LFQ intensity_ = 64), retinol binding protein 3 (Gene name: *RBP3*) (RET/ONH_LFQ intensity_ = 43), and glutamate dehydrogenase 1 (Gene name: *GLUD1*) (RET/ONH_LFQ intensity_ = 37) showed high RET-associated abundance. The lowest relative RET abundances resulting in low RET/ONH LFQ intensity ratios were recorded for voltage-dependent anion-selective channel protein 1 (Gene name: *VDAC1*) (RET/ONH_LFQ intensity_ = 0.7) and 2 (Gene name: *VDAC2*) (RET/ONH _LFQ intensity_ = 0.8), profilin (Gene name: *PFN1*) (RET/ONH_LFQ intensity_ = 0.7), and spectrin ß chain (brain 1) (Gene name: *SPTBN1*) (RET/ONH _LFQ intensity_ = 0.8). Furthermore, GO analysis showed that the RET-specific proteins were mainly represented by intracellular protein species, whereas the ONH-specific proteins were predominantly found intracellularly as well as in the extracellular milieu. This observation is in accordance with the finding that the majority of RET-specific membrane proteins are exclusively associated with organelle membranes, whereas ONH-specific proteins are largely associated with cell membranes as well as organelle membranes. Methyl-CpG-binding protein 2 (Gene name: *MECP2*) could only be recovered in traces from ONH samples, whereas it could be detected with significantly higher abundances in the RET (*p* = 0.005898 [Raw], *p* = 0.000007 [LFQ]; Student’s *t*-test) (Figure 8). Regarding the molecular functions of the differentially distributed proteins in ONH and RET, most proteins are involved in binding processes, whereby no obvious differences towards binding targets could be documented (Figure 9). However, the RET-specific subproteome includes more enzymes than the ONH subproteome (RET = 57%; ONH = 36% GO entries for “catalytic activity”). Tissue-specific proteins could be assigned to a tissue discriminative interaction network by STRING analysis. RET-specific interacting proteins includes predominantly metabolic enzymes, e.g., aconitase hydratase (Gene name: *ACO2*), malate dehydrogenase (Gene name: *MDH2*), aspartate aminotransferases (Gene name: *GOT1* and *GOT2*), glutamate dehydrogenase 1 (Gene name: *GLUD1*), and glutamine synthetase (Gene name: *GLUL*). The ONH subnetwork comprises structural, cytoskeletal proteins, e.g., spectrin (Gene name: *SPTN1*), filamins (Gene name: *FLNB* and *FLNC*), talin 1 (Gene name: *TLN1*), vinculin (Gene name: *VCL*), vimentin (Gene name: *VIM*), tropomyosin (Gene name: *TPM2*), as well as myelin-associated proteins interacting with peptidyl arginine deiminase 2 (Gene name: *PADI2*). Extracellular stress proteins highlighting serotransferrin (Gene name: *TF*), endoplasmin (Gene name: *HSP90B1*), ceruloplasmin (Gene name: *CP*), peroxiredoxin 2 (Gene name: *PRXD2*), and clusterin (Gene name: *CLU*), as well as the regulatory proteins aminopeptidase N (Gene name: *ANPEP*), guanylate cyclase (soluble subunit ß-1) (Gene name: *GUCY1B3*), and signal transducer and activator of transcription 1 (Gene name: *STAT1*) were found as linkage components between the ONH cytoskeletal and RET metabolic networks (Figure 10). In summary, a first impression of the proteomic heterogeneity of two ocular tissues at the interface of neurodegenerative pathologies, e.g., glaucoma, could be documented in the present study.

## 3. Discussion

The ONH represents an interface tissue that distinctly differs from the RET tissue in morphology, cellularity, and function. Whereas retinal ganglion cell (RGC) soma and non-myelinated parts of axons are located within the retina, non-myelinated and initially myelinated axon parts are main integrative ONH components. These cellular and functional differences became obvious on a proteomic scale referring to LC–MS measurements of porcine material presented in the current study. Numerous proteins were documented that display tissue-specific distribution in RET and ONH. Particular proteins could be annotated to neurodegenerative processes which play an important role in glaucoma. Peptidyl arginine deiminase 2 (Gene name: *PADI2*) was found at significantly higher abundance in ONH than in RET samples and was reported to be elevated in human primary open-angle glaucoma (POAG) optic nerve [21,22]. Since the enzyme is significantly more abundant in the ONH interacting with the ONH myelin and cytoskeletal network, a key role for ONH structure and function can be suggested. Aquaporin 4 (Gene name: *AQP4*), primarily found in ONH tissue in the present study, was recovered from fibrous optic nerve astrocytes and retinal Müller cells [23]. Yang and colleagues (2001) [24] reported serum autoantibodies against glutathione S-transferase in glaucoma, whereby the P variant (Gene name: *GSTP1*) was specifically detected in RET samples in the present work. ONH‑specific gelsolin (Gene name: *GSN*), prostaglandin D synthase (Gene name: *PTGDS*), as well as calponin (Gene name: *CNN3*) were reported as glaucoma ONH astrocyte markers [25]. Glutamine synthase (Gene name: *GLUL*), a retinal Müller cell marker, was found as an oxidation target protein in a glaucoma rat model [26] and was shown to be up-regulated in retinal rat Müller cells by hypertension [27]. In addition, GLUL seems to play a key role in the retinal glutamate/glutamine cycle in hypertensive eyes [28]. Glutamate dehydrogenase 1 (Gene name: *GLUD1*) could be found primarily in RET tissue, and both proteins (GLUL and GLUD1) have been associated with neurodegenerative processes in the context of glutamate metabolism in neuronal tissue [29,30]. Methyl-CpG-binding protein 2 (Gene name: *MECP2*), as an important neurodegeneration-associated protein, was primarily recovered from RET samples, most likely reflecting its nucleus residence in RGC soma. Despite the stringent RET localization of MECP2, this protein could be observed at low abundances in few ONH samples, which might indicate subcellular protein traffic in the axons between both tissues (RET and ONH). However, the main residence of MECP2 is clearly restricted to RET, which has to be considered for future studies on MECP2. Expression of RET-recovered malate dehydrogenase (Gene name: *MDH2*), a key metabolic enzyme of the citrate and aspartate cycle, was found to go along with the expression levels of the glutamate/aspartate-converting key enzyme aspartate aminotransferase in the rodent retina [31,32]. This supports the important role of MDH2 in the vertebrate retina. Mitochondrial aconitate hydratase (Gene name: *ACO2*), which was demonstrated to show RET‑specific abundance, was reported as an oxidative stress‑related key enzyme in terms of neurodegeneration [33]. Typical neurodegenerative-associated proteins could be recovered from ONH, comprising neurofilament medium polypeptide (Gene name: *NEFM*) and glial fibrillary acidic protein (Gene name: *GFAP*) [34,35]. Moreover, the interaction analysis revealed a strong connection between RET metabolism and ONH cytoskeleton, highlighting extracellular stress proteins as potential linking components. Oxidative stress-related neurodegeneration-associated proteins such as hypoxia upregulated protein 1 (Gene name: *HYOU1*) [36] or ACO2 [37] underline a RET-specific function with respect to metabolic demand and vulnerability. ONH structure and metabolism are strongly correlated and affected in the course of glaucoma [38]. A potential link between RET metabolism and ONH structure through soluble extracellular stress proteins was shown, which is of interest, considering that strong associations with different glaucoma phenotypes have been reported for extracellular ceruloplasmin (Gene name: *CP*) [39,40] and clusterin (Gene name: *CLU*) [41,42]. Especially CLU is strongly associated with other neurodegenerative disorders [43]. Interestingly, in the present study, CP was exclusively identified in the ONH tissue samples, whereas CLU represents a unique RET‑specific protein marker (see File S1). These tissue-specific expression profiles of both stress markers clearly indicate the complex regulation and interaction between the two posterior ocular tissues (RET and ONH). However, based on the ONH/RET subproteomic interaction network analysis, the cross-talk between ONH-specific stress and cytoskeletal proteins and RET-specific metabolic enzymes has to be addressed in future studies, with special focus on glaucoma research. Talin-1 (Gene name: *TLN1*) could be a promising candidate in this context. It plays a key role in presynaptic function through the interaction with brain-located phosphatidylinositol-(4)-phosphate 5 kinase type Iγ [44] and activates ß-integrin [45]. Integrins are proposed to be translators of mechanical stress [46], also playing a crucial role in the glaucomatous ONH [47]. ONH‑specific TLN1, therefore, is a highly promising protein, which is worth taking a closer look at in future studies on glaucomatous neurodegeneration. Moreover, the expression levels of many other marker proteins such as GFAP [48], heat shock protein 27 (Gene name: *HSP27*) [49], or α-crystallin B chain (Gene name: *CRYAB*) [50] have been correlated with an elevated intraocular pressure (IOP), one of the main risk factors of glaucoma development. They, therefore, represent interesting target molecules and underline the relevance of the house swine as an important model organism for various retinal neurodegenerative disorders [51].

## 4. Materials and Methods

### 4.1. Sample Preparation

RET and ONH tissues were withdrawn from freshly enucleated house swine eye bulbs (*S. scrofa domestica* Linnaeus 1758, 3–6-months old at the date of sacrifice, female/male = 3:2, *n* = 12) provided by a local slaughterhouse (Landmetzgerei Harth, Stadecken-Elsheim, Germany). The approval of the application of animal by-products for research purposes was provided by the Kreisverwaltung Mainz-Bingen in Germany (Identification Code: DE 07 315 0006 21, approved on 13 January 2014). Eye bulbs were transversally cut while cooled on ice. After removing the lens and the vitreous body, the eye cups were flushed gently with cold PBS. RET was removed, and ONH was circularly cut and excised. The tissue samples were immediately snap‑frozen in liquid N_2_ and manually grinded with a 1.8 mm diamond milling head (LUX-TOOLS, Wermelskirchen, Germany). The resulting homogenates were mixed with extraction buffer (0.1% dodecyl-ß-maltoside (DDM), 10% acetonitrile (ACN), tissue weight/extraction volume = 1:1) and incubated in an ultrasonic bath for 10 min in iced water. The samples were centrifuged at 10,000× *g* for 12 min. The supernatants were stored at −20 °C until further analysis. Aliquots of 10 µL per sample were used for protein concentration determination with the Pierce BCA protein assay kit (Thermo Fisher Scientific, Rockford, IL, USA) and measured with the Multiscan Ascent plate reader (Thermo Fisher Scientific, Rockford, IL, USA) at the wavelength of 570 nm.

### 4.2. BULCMS Analysis

Four technical replicates (50 µg/replicate) of each homogenized tissue type (ONH and RET tissue pools were received from 12 eye bulbs, see Section 4.1) were run under reduced conditions on 10-well NuPAGE 12% Bis-Tris minigels (Invitrogen, Carlsbad, CA, USA) with 3-(N-morpholino) propane sulfonic acid (MOPS) buffer at 150 V for 1h. After separation, the gels were fixed and stained using the Novex Colloidal Blue Staining Kit (Invitrogen, Carlsbad, CA, USA). The gels were scanned on a DCP-9042 CDN bench top scanner (Brother Industries Ltd., Nagoya, Japan) at 1200 × 1200 dpi. Gel lanes of each tissue (ONH and RET, *n* = 4) were sliced with respect to their specific protein distribution pattern followed by in-gel trypsin digestion using a modified protocol of Shevchenkov and coworkers (2006) [52]. After peptide solid-phase extraction (SPE) purification with C18 ZIPTIP^®^ pipette tips (Millipore, Billerica, MA, USA) [12], 10 µL of 0.1% trifluoroacetic acid (TFA) peptide aliquots were used for LC–MS/MS analysis. The LC–MS system consisted of a Rheos Allegro (Thermo Fisher Scientific, Rockford, IL, USA) capillary HPLC system (BioBasic C18 pre [30 × 0.5 mm] and analytical column [150 × 0.5 mm]; flow rate: 6.7 ± 0.03 μL/min flow) (Thermo Fisher Scientific, Rockford, IL, USA). Each run corresponded to an injection of 5 µL of peptide solution and a 50 min gradient elution (buffers: A = 98% H_2_O, 1.94% ACN, 0.06% methanol, 0.05% formic acid; B = 95% ACN, 3% methanol, 2% H_2_O, 0.05% formic acid; gradient program: 15–20% B (0–2 min), 20–60% B (2–35 min), 60–100% B (35–40 min), 100–0% B (40–45 min), 0% B (45–50 min)]. Proteomic raw data were recorded on an LTQ Orbitrap XL MS hybrid instrument (Thermo Fisher Scientific, Rockford, IL, USA) in a mass range of 300–2000 *m/z*. The following mass detection parameters were set: 50 ms LTQ injection time, 500 ms FT injection time, Lock Mass correction [35], collision-induced decay normalized energy of 35, 30 ms activation time, 0.25 activation Q, 2 *m/z* isolation width for fragmentation, dynamic exclusion of 90 s, repeat duration of 30 s, resolution of 30,000, centroid detection, fragmentation selection of top 5 monoisotopic *m/z* signals (z = 1–4+, intensity > 500). The system is well established in proteomic studies on neuroretinal cells [53], retinal tissues [10,12,54], and human serum samples [55]. Raw data were subjected to MaxQuant analysis (version 1.4.1.2 ; Max Planck Institute of Biochemistry, Martinsried, Germany; www.maxquant.org) for protein identification and label-free quantification [56] considering Swissprot pig protein database (uniprot_SusScrofa_Canonical _Isoforms_150911.fasta). Mass tolerances were adjusted to 30 ppm (precursor peptides) and 0.5 Da (fragments). Carbamidomethylation (C) was set as fixed modification, oxidation (M) and acetylation (protein N-term) were set as variable modifications. Trypsin was chosen as the cutting enzyme, and two missed cleavages per peptide were allowed. For stringent identification, protein output was confidently filtered based on false discovery rate (FDR) < 1%. A minimum ratio count of two, considering at minimum one razor peptide (≥6 amino acids) was set for quantification. Both MaxQuant-specific RAW and LFQ-normalized peak intensities were transferred to Statistica version 10 (Statsoft, Tulsa, OK, USA), and unpaired *t*-test statistics was performed.

### 4.3. MECP2 Targeted Detection

Methyl-CpG-binding protein 2 (Gene name: *MECP2*), a neuronal nucleus protein associated with neurodegeneration [57,58], was previously shown to display glaucoma-related alterations in human retinal tissue samples [13]. Thus, the aim of our investigation was to determine tissue-specific differences in the abundance of MECP2 between RET and ONH. The extraction of the target protein MECP2 is routinely performed by using acidic extraction buffers, as described in a previous study [10], and was applied to the post-extraction tissue pellets (see Section 4.1). Therefore, in the remaining pellets containing the organelle debris incorporating nucleus proteins, MECP2 was quantified in a label-free manner using the AIMS strategy [59]. The RET and ONH post-extraction pellets of six eye bulbs were extracted twice with two volumes of pellet extraction buffer (50 mM ammonium bicarbonate, 20% ACN, 1% TFA) with respect to pellet weights. After centrifugation (10,000× *g*, 4 °C, 10 min), the corresponding supernatants were combined and adjusted to pH 7.4 with 500 mM ammonium bicarbonate buffer, and a subsequent BCA assay for protein concentration determination was performed. The extraction procedure was realized in duplicate (*n* = 2 biological replicates). For in‑solution trypsin digestion, 10 µg of RET or ONH pellet extracts of each replicate was mixed with 2 µg Sequencing-Grade Trypsin (enzyme/protein = 1:50) and incubated overnight at 37 °C. Peptide SPE purification was performed using the already described C18 ZIPTIP^®^ protocol [12]. The resolubilized peptide samples were analyzed utilizing the described LC–ESI-MS/MS workflow in combination with the targeted AIMS strategy. The relative concentration of MECP2 was determined in RET and ONH tissues (from *n* = 6 eye bulbs) after two extraction steps (*n* = 2 biological replicates for each sample). Thereby, MECP2 identification and label-free quantification were based on monitoring three unique *MECP2* reporter peptides (*VGDTSLDPNDFDFTVTGR* [978.45 *m/z*, *z* = 2, MH+ = 1954.89 Da], *KAEADPQAIPK* [584.32 *m/z*, *z* = 2, MH+ = 1166.63 Da], *SKVELIAYFEK* [663.87 *m/z*, *z* = 2, MH+ = 1325.73 Da]), using MaxQuant software package considering the previously described LC–MS settings (see Section 4.2).

### 4.4. MALDI-TOF MS Analysis

In-solution trypsin digestion of RET (*n* = 12) and ONH (*n* = 12) tissue samples was performed (50 µg /sample; trypsin/protein amount = 1:10). A total protein amount of 5 µg per resulting peptide sample was acidified (0.1% TFA) and incubated with 29 µL conditioned magnetic RPC18 Dynabeads (Invitrogen, Carlsbad, CA, USA), followed by stepwise elution in four 6 µL fractions (0.1% TFA containing 20, 30, 40, and 50% ACN). The peptides were lyophilized and resolubilized in 0.1% TFA. Five replicates of each peptide fraction (2 µL/replicate) were spotted on a 386 MTP polished steel MALDI target plate (Bruker Daltonics, Bremen, Germany) and air-dried, followed by matrix application (19 mg of α-4-cyano-hydroxy cinnamic acid, 60% ACN; 2% TFA; 2µL/spot). Analysis was performed using an Ultraflex II MALDI-TOF–TOF MS analyzer (Bruker Daltonics, Bremen, Germany) equipped with a nitrogen laser in the instrument’s reflector mode. MS data were recorded in a detection range of 900–2200 *m/z* considering a signal-to-noise ratio of 6. Thereby, 500 laser shots in 50 units were accumulated for each MS spectrum with fuzzy controlled laser power (40–80%). MS data were externally calibrated using the peptide II calibration standard (Bruker Daltonics, Bremen, Germany) and internally calibrated considering autodigestive trypsin peaks. MS data inspection was realized in Flex Analysis version 2.4 (Bruker Daltonics, Bremen, Germany). Peptide reporter peaks of 27 protein candidates revealed from the BULCMS analysis were selected for MALDI-specific fragmentation. For this purpose, an in‑house established pig retina MALDI reporter peak reference list within a 20 ppm tolerance window was created and subjected to further MALDI-TOF–TOF MS/MS post-source decay fragmentation (PSD) experiments using BioTools software (version 3.0; Bruker Daltonics, Bremen, Germany). Semi-quantitative determination was realized on the raw intensities of monoisotopic SNAP-detected peptides of specific protein candidates among the technical replicates. Statistical unpaired *t*-tests of protein levels were performed using Statistica version 10 (Statsoft, Tulsa, OK, USA).

### 4.5. Functional Analysis

Tissue-specific protein candidates were scheduled for gene ontology (GO) analysis using Cytoscape version 2.8.3 with the implemented BINGO 2.44 plugin (www.cytoscape.org). Protein‑protein interactions were determined using STRING version 10 (Search Tool for the Retrieval of Interacting Genes/Proteins), considering a medium confidence (score 0.4) for interactions.

## 5. Conclusions

In conclusion, the presented study highlights the proteomic heterogeneity of RET and ONH, which indicates an adaption to different ocular functions, emphasizing the association of RET metabolism and ONH structure on a proteomic scale. Moreover, the ONH/RET proteomic catalogue can provide an important reference in terms of ocular porcine disease models including neurodegeneration and proposes numerous tissue-specific protein candidates with a high potential for future studies in the field of glaucoma- and neurodegeneration-related research.

## Supplementary Materials

Supplementary materials can be found at https://www.mdpi.com/1422-0067/20/17/4229/s1. File S1: Protein identifications from the retina (RET) and the optic nerve head (ONH) as well as biomarker verification results by MALDI-TOF MS (MB analysis).
